# WHAT’S ON THE CALENDAR THIS WEEK? EVERYDAY COLLABORATIVE STRATEGY USE AMONG MIDDLE-AGED AND OLDER COUPLES

**DOI:** 10.1093/geroni/igad104.2633

**Published:** 2023-12-21

**Authors:** Celinda Reese-Melancon, Rachael Turner, Riley Wyatt, Erin Harrington, Tina Savla, Peter Rendell, Jennifer Margrett

**Affiliations:** Oklahoma State University, Stillwater, Oklahoma, United States; Oklahoma State University, Stillwater, Oklahoma, United States; Oklahoma State University, Stillwater, Oklahoma, United States; University of Wyoming, Laramie, Wyoming, United States; Virginia Tech, Blacksburg, Virginia, United States; Australian Catholic University, Melbourne, Victoria, Australia; Iowa State University, Ames, Iowa, United States

## Abstract

Mnemonic strategies support individual memory performance in a variety of domains, including school, work, and home. Less is known about how people collaborate to remember information in their daily lives. We asked married and/or cohabitating middle-aged and older adults (118 individuals, M age = 62 years, Range = 40-88 years, 50% female) about their individual strategy use and whether they engaged in any collaborative behaviors to support their everyday memory success. We employed a mixed methods approach to data analysis. We found that, as a group, husbands did not statistically differ from wives in their reports of individual internal, external, or technology-based strategy use. Most participants did report collaborating with their partner to remember the things they need to do in a given week. Themes regarding collaborative strategy use included weekly or daily calendar planning, making joint To-Do lists or making such a list for one’s partner, conferring about upcoming events and appointments, and providing reminders to partners. These findings lay the groundwork for future work examining how partners may support each other cognitively as they age and whether these supports change over time.

